# Adipokines, Cardiovascular Risk, and Therapeutic Management in Obesity and Psoriatic Arthritis

**DOI:** 10.3389/fimmu.2020.590749

**Published:** 2021-02-10

**Authors:** Sabrina Porta, Matilde Otero-Losada, Rodolfo A. Kölliker Frers, Vanesa Cosentino, Eduardo Kerzberg, Francisco Capani

**Affiliations:** ^1^ Rheumatology Department, J. M. Ramos Mejía Hospital, Buenos Aires, Argentina; ^2^ Biomedical Research Center, Interamerican Open University, National Research Council (CAECIHS-UAI. CONICET), Buenos Aires, Argentina; ^3^ Department of Biology, University John F. Kennedy, Buenos Aires, Argentina; ^4^ Universidad Autónoma de Chile, Santiago, Chile

**Keywords:** psoriatic arthritis, metabolic syndrome, cardiovascular risk, obesity, adipokines, pathophysiology, treatment

## Abstract

Psoriatic arthritis is a chronic inflammatory disease with skin and joint pathology as the dominant characteristics. Scientific evidence supports its systemic nature and relevant relationship with obesity, metabolic syndrome, and associated conditions. Metabolic syndrome and obesity share common signaling pathways with joint inflammation, reinforcing the idea that adipose tissue is a major contributor to disease development and severity. The adipose tissue is not a mere energy store but also an endocrine organ participating in the immune response. In the search for the best therapeutic strategy for a patient, we should appraise the adipose tissue as an endocrine and immune organ responsible for mild chronic inflammation. Today, our challenge is not only to achieve disease remission but to control the associated comorbidities as well. In light of the high prevalence of obesity in psoriatic arthritis patients and the importance of the adipose tissue in the development of chronic inflammation, we aimed to identify the most relevant articles in this regard published in English until June 2020 using the PubMed database. Search terms included psoriatic arthritis, in combination with metabolic syndrome, obesity, adipokines, cardiovascular disease, and treatment. This review summarizes the current evidence regarding the role of adipose tissue as an adipokine-secreting endocrine organ, discussing its influence on disease development and severity, and ultimately in meeting successful disease management.

## Introduction

Psoriasis is a chronic inflammatory disease mainly compromising skin with systemic involvement associated with various comorbidities (1). With 6% to 40% percent of psoriatic patients experiencing psoriatic arthritis (PsA) (2), it equally affects men and women, starting usually at the age of 40 (3). The first signs appear on the skin usually 10 years before arthritis is diagnosed (4, 5).

Rheumatoid factor and cyclic citrullinated anti-peptide are absent, so PsA is considered a seronegative arthritis. Five PsA phenotypes may be distinguished based on the articular compromise pattern: predominantly axial, symmetric polyarthritis, asymmetric oligoarthritis, predominantly distal interphalangeal damage, and mutilating arthritis (6, 7).

Two distinguishing features of the disease are inflammation at the tendon and ligament insertions in the bone or enthesitis, and dactylitis when inflammation affects one finger entirely, risking the tendon sheath (8, 9).

The diagnosis of PsA is mainly clinical, reckoning on the classification criteria for psoriatic arthritis (CASPAR) (10, 11) (CASPAR) (10, 11).

Besides synovitis, enthesitis, dactylitis, and spondylitis, PsA typically presents non-musculoskeletal manifestations as anterior uveitis and inflammatory bowel disease. Comorbidities like obesity, cardiovascular disease, metabolic syndrome (MetS), and its components (obesity, diabetes mellitus, hypertension, and dyslipidemia) are also more frequent than in the general population (12).

Although the etiopathogenesis of PsA is multifactorial and not entirely known, there is an interaction between genetic susceptibility factors, mainly human leukocyte antigens (HLA) B27, together with other HLA-B loci and the HLA-Cw * 0602 allele) and environmental triggers (such as mechanical stress, dysbiosis, trauma, smoking, or infection), leading to dysregulation of immunoinflammatory pathways and the development of the disease (3).

In the last decades, a substantial body of evidence indicates that cutaneous psoriasis and psoriatic arthritis patients are at higher risk of developing cardiovascular disease. It is known that adipose tissue is metabolically active and an important source of inflammatory adipokines. Obesity and atherogenesis are low-grade inflammatory diseases and a link with associated PsA. Inflammatory mediators of obesity may represent one added key cardio-metabolic risk factor in PsA subjects (13).

The prevalence of immune-mediated diseases like psoriasis has increased in industrialized countries in the last decades, likely accountable to environmental factors, considering that the genetic predisposition has not changed (14). Western lifestyle, sedentary, with high fat and carbohydrate diets, and excessive sodium consumption favor the development of overweight and obesity (15).

In light of the high prevalence of obesity in psoriatic arthritis patients (16) and the importance of the adipose tissue in the development of chronic inflammation, we aimed to identify the most relevant articles in this regard published in English until June 2020 using the PubMed database. Search terms included psoriatic arthritis, in combination with metabolic syndrome, obesity, adipokines, cardiovascular disease, and treatment.

This review summarizes the current understanding of the role of the adipose tissue as an adipokine-secreting endocrine organ, discussing its influence on disease development and severity. Ultimately, robust evidence acknowledges considering the relevance of the adipose tissue in meeting successful disease management.

## Pathogenesis of Psoriatic Arthritis

Multiple factors participate in PsA development in an as yet not fully understood fashion. Some environmental factors like infections or mechanical stress give way to a genetically predisposed terrain, activating both the innate and adaptive immune systems. Genetic variants the major histocompatibility complex molecules and inflammatory mediators like the IL-12B gene, which encodes the p40 subunit of IL-23 and IL-12, the IL-23A gene, encoding for the p19 subunit of IL-23, and the IL-23 receptor gene (IL-23R), which encodes a common domain of IL-23R and IL-12R could favor the IL-23/IL17 axis activation, predisposing to the disease (17).

Chronic infections have raised great interest. In dysbiosis, microbiome proliferation, distorting its balance with the immune system, aberrant activation of the latter could favor cells recruitment, T helper (Th) 17 lymphocytes, in particular (17, 18). Activated Th17 lymphocytes might migrate from the intestinal mucosa and lymph nodes to the skin and joint tissue, causing local inflammation.

Repeated trauma causes tissue damage at the enthesis, i.e., tendon, ligament, and joint capsule insertion to the bone, activating the innate immune system by Damage-Associated-Molecular-Patterns (DAMPs) recognition (17, 19).

The growing importance of entheses in PsA pathophysiology has encouraged research studies. The enthesis is no longer considered a simple focal insertion but referred to as the ‘organ of enthesis’ (20). A healthy enthesis is usually anti-inflammatory, unlike the synovial membrane, which is proinflammatory regarding cellular composition and structure. When a mechanically stressed enthesis is injured, the associated inflammatory reaction manifests prominently within the juxtaposed synovium. The “synovio-entheseal complex” concept supports that specific factors at the joint level are relevant as danger signals, activating innate immune responses (20).

Regarding the adaptive immune system, IL17 A and IL23 appear key in PsA development.

Interleukin-17 not only activates innate-immunity cells but epithelial and endothelial cells, keratinocytes, chondrocytes, osteoblasts, and osteoclasts as well. Its ability to increase the level of IL1-IL6, IL8, TNF-α, matrix metalloproteinase-9 (MMP-9), granulocyte-macrophage colony-stimulating factor (GM-CSF), inducible nitric oxide synthase (iNOS), and receptor activator of nuclear factor-kappa B (RANK) applies to its participation in the inflammatory cascade (17).

In psoriatic patients, IL-17 handles CCL2 aberrant expression on keratinocytes, which recruits inflammatory cells, leading to hyperkeratosis and cell dysfunction (17).

Interleukin-17 and IL-23 act synergically in joint injury development, playing complementary roles in PsA pathogenesis. The respective intracellular signaling cascades are different, although IL-23 activates Th17 lymphocytes.

Antibodies against the keratinocyte cytoskeleton and enthesis sites have been identified, posing new theories as to disease development (21), while TH2-type response participation in seronegative arthritis has not been classically considered.

### Psoriatic Arthritis and Cardiovascular Risk

Patients with PsA have increased cardiovascular risk, while the debate about the compromised metabolic pathways remains open (22). The so-called traditional cardiovascular risk factors, as hypertension, obesity, smoking, dyslipidemia, and metabolic syndrome, and hence the cardiovascular risk are increased in PsA patients (23, 24). Psoriasis is a systemic chronic inflammatory condition, where a high level of pro-inflammatory cytokines spreads beyond skin limits. Then, endothelial damage might be responsible for the increase of atherosclerotic disease in PsA patients (22). The ‘two plaques for one syndrome’ theory has been put forward. It states that a similar cytokine profile and inflammatory infiltrate is found in plaques both in skin psoriasis and atheroma (25).

We reported evidence supporting the contribution of classic cardiovascular risk factors and systemic inflammatory milieu in disease development. We found an increased level of C-reactive protein (CRP) and soluble intercellular adhesion molecule-1 (sICAM-1)in PsA patients without cardiovascular history or traditional risk factors compared with healthy subjects, the same as in recent-onset PsA patients, in the absence of cardiovascular risk factors (13).

### Psoriatic Arthritis and Metabolic Syndrome

Psoriatic arthritis is undeniably a systemic disease, well expressed by the concept of psoriatic disease, having joint compromise as the central and most relevant characteristic (26). Within the spectrum of extra-articular manifestations, MetS stands out. Over the years, the diagnostic criterion for MetS, a cluster of coexistent hypertension, obesity, insulin resistance, and dyslipidemia, has undergone changes, each of which posing per se cardiovascular risk (27) Joint inflammation and MetS share common signaling pathways, which result in cardiovascular disease (3, 28). Atherosclerosis, MetS, and PsA show a common pattern of T-cell activation. They increase cytokine production with a Th-1 profile as tumor necrosis alpha (TNF-α), interleukin 1 beta (IL1-β), interleukin 10 (IL-10), and interferon (IFN) (3, 29). These cytokines induce insulin resistance in skeletal muscle, increase hepatic synthesis of pro-coagulant factors, inhibit lipoprotein lipase, and increase fatty acid oxidation, and atherogenic lipoprotein serum level. Cytokine production, insulin resistance, and endothelial dysfunction favor the formation and deposition of atheroma plaques in a deleterious positive feedback cycle (30). Proinflammatory cytokines not only promote vascular wall recruitment of macrophages and T lymphocytes precipitating atheroma plaque formation and deposition but induce foam cell lysis. This rupture or plaque accident releases cellular debris and thrombi to circulation with the consequent at distance damage often presented as acute myocardial infarction and cerebrovascular accident. The relationship between joint inflammation and atherosclerosis has led to considering PsA a cardiovascular risk factor on its own (31). Furthermore, PsA patients having no cardiovascular risk factors showed increased carotid intima-media thickness (IMTc) compared with control subjects (32, 33). In 2015, Di Minno et al.’s meta-analysis reported a higher carotid IMT and prevalence of carotid plaques, and reduced flow-mediated dilatation in 898 PsA patients compared with 1140 control (34). Although lifestyle changes are the fundamental pillar in MetS management, pharmacological intervention is often necessary. However, confirmed PsA diagnosis should refocus the attention to the inflammatory activity since psoriasis intrinsically favors endothelial dysfunction, insulin resistance, and a prothrombotic environmental background. Accordingly, the adequate MetS control should also consider joint disease remission.

### Psoriatic Arthritis and Obesity

The complex, multifactorial relationship between obesity and psoriasis is not clear. Common pathophysiological mechanisms might account for pinning a vicious cycle that results in both. Whether one causes the other or they express different aspects of a same disorder is still undetermined.

While well-known factors may be accountable for arthritis development in PsA patients like severe skin compromise, nail compromise, genetic predisposition, and obesity, the latter has the advantage that can be changed.

Functional disability in PsA reduces physical activity, favoring weight gain, and, conversely, obesity prior to arthritis development is also true. This supports that chronic inflammation is likely involved in PsA development.

Two hypotheses explain how obesity favors PsA development in genetically predisposed subjects (35). One hypothesis proposes that adipose tissue acts as a source of inflammatory mediators like adipokines and proinflammatory cytokines, including TNF-alpha and IL-6 (30). The other one puts forward that overweight might would stress the enthesis due to the increase in the mechanical load with the following microtrauma, enticing an aberrant inflammatory response and PsA development (20). Obese patients had a higher prevalence of tuft resorption, Achilles and calcaneal spurs, and pelvic enthesitis compared with normal-weight subjects, reinforcing this hypothesis. Obesity was related to a late PsA onset, while normal-weight was associated with the HLA-B27 allele and an earlier onset of the disease (36).

Proinflammatory cytokines increased in obese patients are particularly involved in the pathophysiology of PsA and other inflammatory diseases.

Obesity is related to systemic inflammation and shares several pathways with PsA. Obesity, inflammatory status, and PsA are definitely related. A high level of inflammatory adipokines as found in obese patients may favor PsA expression in predisposed people.

**Table 1** shows most of the few publications addressing the role of adipokines in PsA.

**Table 1:** Representative studies linking PsA with adipokines published between 2009 and 2019.

	Objective and characteristics	Main results
Eder et al. (37)	To compare MetS prevalence and levels of related biomarkers between 203 PsA patients and 155 controls.	MetS was higher in PsA (36.5%) compared with PsC (27.1%) p=0.056). Adiponectin was significantly associated with PsA (p=0.005), the use of anti-tumour necrosis factor α therapy (p=0.03) and active joint count (p=0.001).
Feld et al. (38)	To compare the prevalence of MetS and levels of related biomarkers between 74 PsA patients and 82 control subjects in a Mediterranean population.	MetS was higher in PsA patients compared with the control group: 54.8% versus 36.6%, respectively (P = 0.02). Leptin levels and leptin/adiponectin ratio were higher in PsA patients compared with controls: 83.4 versus 51.7 ng/mL (P = 0.001) and 6.3 × 10^−3^ versus 4.1 × 10^−3^ (P = 0.015), respectively.
Xue et al. (39)	To examine TNF-α, OPG, RANKL, leptin, adiponectin, resistin, chemerin, and omentin in 41 PsA patients, 20 PsO patients, and 24 healthy controls.	Compared with healthy controls, PsA patients had higher TNF-α, RANKL, OCs, leptin, and omentin, but lower adiponectin and chemerin.Increased serum levels of TNF-α, RANKL, leptin, and omentin were positively correlated with OCs numbers. Adiponectin level negatively correlated with OCs numbers. TNF-α, RANKL and leptin were positively correlated with disease activity.Only TNF-α was positively correlated with radiographic damage scores.
Caso et al. (40)	To investigate possible differences and correlations between adipokines and clinical expression in 42 PsA patients with clinically evident psoriasis (group 1) and 38 PsA patients without psoriasis (group 2)	A positive association was shown between leptin levels and female sex (β = 0.3, p = 0.001), BMI (β = 0.8, p < 0.0001), tender joint count (β = 0.23, p = 0.05), and patient pain-VAS score (β = 0.4, p = 0.049). In group 1, serum concentration of leptin was associated with female sex (β = 0.41, p < 0.0001) and BMI (β = 0.6, p = 0.012), whereas in group 2, a positive association was shown between leptin levels and BMI (β = 0.7, p = 0.003) and CRP (β = 0.35, p = 0.012). With regard to resistin, in the multivariate model, only the association between resistin and IL-6 was found (β = 0.33, p = 0.002). The association between resistin and IL-6 was confirmed in group 1 (β = 0.46, p = 0.004) but not in group 2.
Dikbas et al. (41)	To examine serum levels of adiponectin, resistin and visfatin, and their associations with disease activity and insulin resistance in 28 PsA patients and 39 healthy controls.	Levels of adiponectin, resistin and visfatin were higher in PsA patients compared with healthy controls (P < 0.05). Adiponectin (P = 0.001, OR = 3.1, 95% CI = 1.6–6.0), resistin (P = 0.06, OR = 1.8, 95% CI = 1.2–2.9) and visfatin (P = 0.03, OR = 3.9, 95% CI = 1.1–13.9) may contribute to pathogenesis of PsA.
Peters et al. (42)	To examine the effect of TNFα blockade therapy on adiponectin in 171 patients with rheumatoid arthritis (RA).	The mean ± SD absolute change in adiponectin levels was −0.23 ± 4.6 μg/ml in PsA patients treated with combined onercept 50 mg and onercept 100 mg (vs placebo, p=0.60) and 0.28 ± 3.23 μg/ml in RA patients treated with adalimumab (vs baseline, p=0.66).
Fassio et al. (43)	To evaluate secukinumab effect on different adipokines in 28 PsA patients.	In the male group, both resistin (p= 0.016) and chemerin (p= 0.028) showed a significant decrease compared with baseline after 6 months of therapy. A positive correlation was found for the overall values of CRP with resistin (p<0.001, R^2 =^ 0.326) and chemerin (p<0.001, R^2 =^ 0.251), and a weak negative correlation for adiponectin (p<0.046, R^2 =^ 0.036).
Chandran et al. (44)	To compare markers of cartilage metabolism, MetS and inflammation in 201 OA patients, 77 PsA patients, and 76 controls.	Levels of resistin, HGF, insulin, leptin, CRP, IL-6, IL-8, TNF-α, MCP-1, NGF were varied across the three groups (p<0.001). In multivariate analysis, resistin (OR = 1.26, 95% CI = 1.07 to 1.48), MCP-1 (OR = 1.10, 95% CI = 0.07 to 1.48), and NGF (OR < 0.001, 95% CI = <0.001 to 0.25) were independently associated with PsA vs OA.
Colak et al. (45)	To compare the relationship between disease activity and vaspin, neutrophil gelatinase-associated lipocalin (NGAL), and apolipoprotein levels in 50 PsA patients and 36 healthy controls.	The levels of vaspin, (391.63 ± 436.4 vs 176.67 ± 122.75, p = 0.001), NGAL (5.2 ± 2.67 vs 1.94 ± 2.09, p = 0.014), and apolipoprotein B/A1 ratio (0.78 ± 0.21 vs 0.66 ± 0.27, p = 0.023) were higher in PsA patients. Apolipoprotein A1 was lower in PsA patients compared with healthy controls, p= 0.017). Patients with MetS had s higher NGAL, Apo B, and Apo B/A1 ratio (p < 0.05). NGAL levels were negatively correlated with disease duration and psoriatic arthritis (p < 0.05). There was a positive correlation for Apo B, Apo B/A1 and BMI with WC (p < 0.05). Patients with MetS had higher scores of DAPSA and PASI (p < 0.05).
Wagner et al. (46)	To determine serum biomarker associations with clinical response to golimumab treatment in the first 100 PsA patients at baseline, week 4, and week 14.	A smaller subset of proteins (adiponectin, apolipoprotein CIII, serum glutamic oxaloacetic transaminase, and TNF α) was associated with a 75% improvement in the psoriasis area and severity index score (PASI75) at week 14,
Johnson et al. (47)	To compare serum biomarkers in 143 PsA patients with 180 PsO.	The level of TNF-α was higher in PsA patients compared with PsO (P < 0.001). A high TNF-α level was associated with increased odds of PsA (multivariate adjusted OR = 2.25, 95% CI = 1 41–3, p = 61). Patients with PSA showed a mild decrease in median total adiponectin and high molecular-weight (HMW) adiponectin. An inverse association was found between high total adiponectin and PsA (multivariate adjusted OR = 0.61, 95% CI = 0.39–0.96). An inverse association of PsA with total or HMW adiponectin was found only in participants reporting alcohol intake.

HOMA-IR, Homeostatic Model Assessment for Insulin Resistance; OPG, osteoprotegerin; RANKL, receptor activator of the nuclear factor-kB ligand; OCs, osteoclast precursors; PsAJAI, Psoriatic Arthritis Joint Activity Index; PASI, psoriasis area and severity index score; HMW, high molecular-weight; RA, rheumatoid arthritis; HGF, hepatocyte growth factor; NGF, nerve growth factor; MCP-1, monocyte chemoattractant protein 1; OA, osteoarthritis; DAPSA, Disease Activity for Psoriatic Arthritis; NGAL, neutrophil gelatinase-associated lipocalin; Apo, apolipoprotein; WC, waist circumference; PsO, poriasis; PsA, psoriatic arthritis.

Despite certain aspects to be clarified, we can affirm that not only psoriasis increases the risk of developing obesity but also obesity is associated with higher prevalence and severity of psoriasis, even in the pediatric population (48, 49).

Studying 943 patients diagnosed with cutaneous psoriasis (CPs) showed BMI at 18 years was predictive of PsA. In the United Kingdom, the risk of developing PsA was studied based on the relationship between the BMI measured after the diagnosis of PsC and PsA development.

Compared with PsC patients with a BMI <25, those with a BMI of 25–29.9, 30.0–34.9 and ≥35.0 had age and sex-adjusted RRs of 1.1, 1.24, and 1.52, (p for trend <0.001) (50). One 14-year follow-up prospective study showed that BMI, weight change from early adulthood, waist circumference, hip circumference, and waist-hip ratio were associated with an increased risk of developing PsA in all the participants and in PsA women in particular (51).

**Figure 1:**
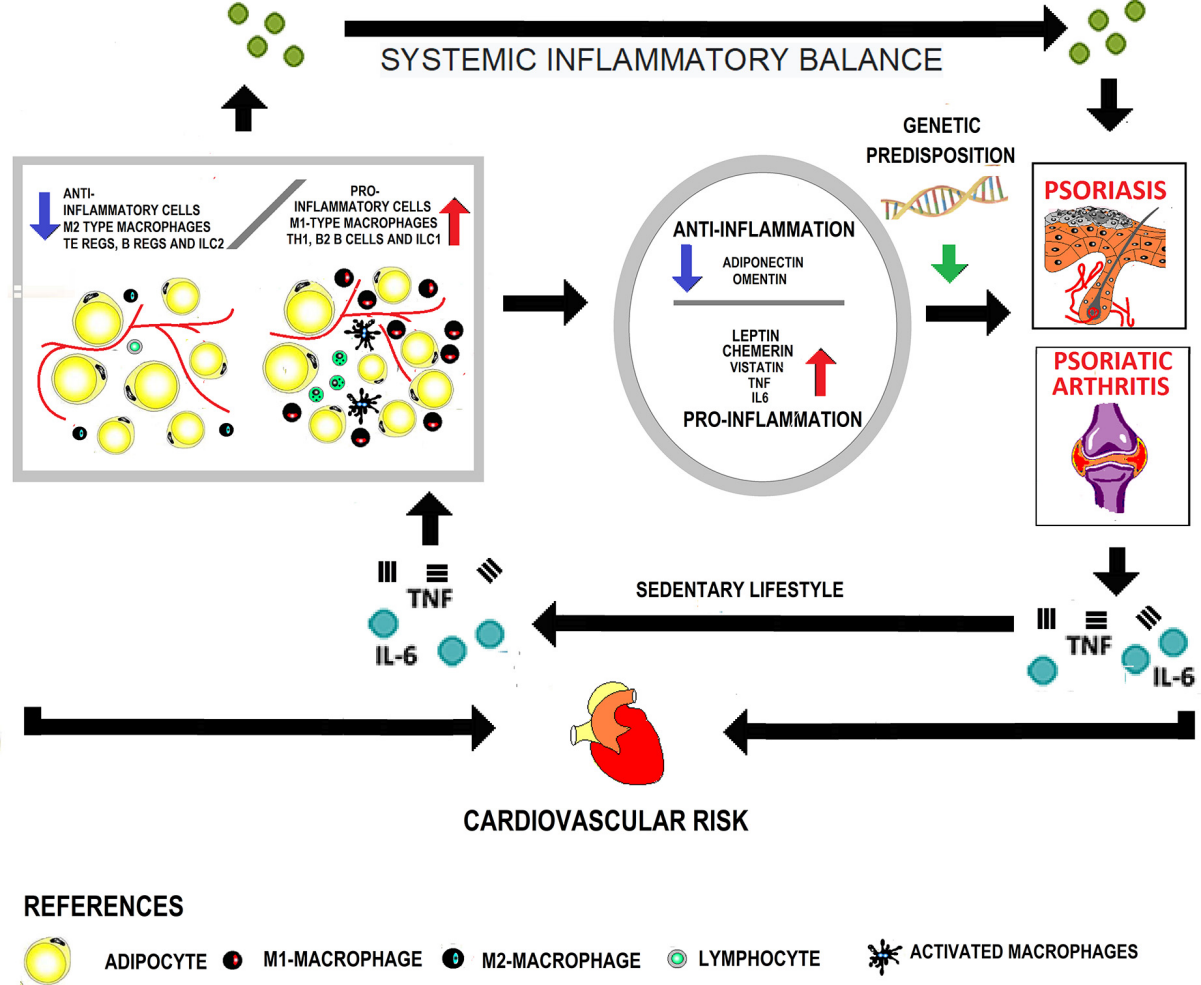
Interaction between obesity and adipokines in the development of psoriasis, psoriatic arthritis, and cardiovascular disease. In obese patients, the adipose tissue presents cellular changes due to greater infiltration of pro-inflammatory cells with a decrease in the anti-inflammatory cell population. In this way, it becomes dysfunctional and acts as an endocrine organ, increasing the secretion of pro-inflammatory adipokines. A systemic inflammatory state is generated that, in genetically predisposed subjects, favors the appearance of skin psoriasis. On the other hand, the increased mechanical load at the enthesis sites contributes to the onset of psoriatic arthritis. The inflammation characteristic of the disease added to the sedentary lifestyle secondary to joint involvement perpetuates the growth of adipose tissue. Finally, both due to the presence of classical and non-classical factors associated with obesity, cardiovascular risk increases.

**Figure 1** shows the interaction between obesity, adipokines, in the development of psoriasis and psoriatic arthritis and cardiovascular disease.

## Characteristics, Distribution, and Function of the Adipose Tissue

Obesity is considered as having a body mass index (BMI) above 30 kg/m2 (52). Its deleterious impact on health has been widely studied, often embedded in the well-known MetS. Metabolic syndrome is a cluster of risk factors leading to microvascular dysfunction and chronic cerebral hypoperfusion, brought by the coexistence of diabetes type 2 or insulin resistance, abdominal obesity, hypertension, and dyslipidemia, mainly a low level of high-density lipoprotein-cholesterol, (HDL) (53–55).

Although weight gain and a high BMI imply an increase in adiposity, studying fat distribution is crucial given the many functions of adipose tissue.

Two types of adipose tissue may be recognized based on cellular structure, location, color, vascularity, and function. Newborns have brown adipose tissue, which has multilocular adipocytes with a large number of mitochondria. Brown adipose tissue is vital at this life stage when heat production is essential for survival. Adults show only white adipose tissue, which is basically an energy store made up of triglycerides within adipocytes (54).

The white adipose tissue presents different distribution patterns, traditionally named as android and gynoid. Android phenotype typically has fat centrally distributed mainly in the abdomen, chest, shoulder, and nape of the neck. Gynoid fat distribution refers to fat peripherally accumulated mainly at the buttocks and hips (54).

The android or gynoid distribution pattern depends on the subcutaneous adipose tissue which contributes to regulate body temperature acting as a thermal insulator.

However, there is another fraction of the white adipose tissue, the visceral adipose tissue, which occupies the space between the internal organs, allowing their proper juxtaposition.

There are important differences between subcutaneous and visceral fatty tissue, because of the expression of different genes related to insulin resistance, inflammation, and a different pattern of adipokines secretion (56).

These different secretion patterns have local repercussions involving autocrine or paracrine mechanisms and systemic ones. In this way visceral fat directly affects the liver, its increase being responsible for the development of insulin resistance, dyslipidemia, glucose intolerance and cardiovascular hypertension (54).

### Adipose Tissue as an Endocrine and Immune-System Organ

Adipose tissue is made up not only of adipocytes which represent one-third of total fat. Adipose tissue also comprises fibroblasts, macrophages, stroma cells, and pre-adipocytes (54) Then, adipose tissue is not a mere energy store but also an endocrine organ participating in the immune response (57).

Adipose tissue is responsible for synthesizing adipokines which form a heterogeneous group including hormones like leptin and adiponectin, cytokines like TNF-α, IL -6, omentin, visfatin, and other proteins like the plasminogen activator (PAI) -1, angiotensinogen and resistin.

The adipokines family includes anti-inflammatory and proinflammatory adipokines. Under normal metabolic conditions, they are balanced. However, their plasma profile changes in response to different challenges, like metabolic variations. A deficit in calorie supply during starvation causes that proinflammatory adipokines decrease, and anti-inflammatory adipokines increase.

### Proinflammatory Adipokines

The hormone leptin is produced mainly by adipocytes and regulates whole-body energy homeostasis. Fat mass and inflammatory status determine leptin levels (30).It reduces food intake and increases energy expenditure, acting directly on the hypothalamus and other brain regions (58). Obese patients typically show a high circulating level of leptin and leptin resistance (57) Leptin pro-angiogenic properties have been described directly related to endothelial dysfunction and atherosclerosis development (59). Leptin also modulates the immune response as an inflammatory molecule capable of activating adaptive immunity cells (57).

High level of leptin found in PsA patients, considering leptin properties, has suggested that leptin might be involved in bone erosion, activating osteoclasts in these patients (22) perhaps due to its cytokine-like structure (30). Leptin correlates with metabolic syndrome features and inflammation in patients with moderate-to-severe psoriasis (60).

Chemerin is involved in inflammation, adipogenesis, angiogenesis, and dyslipidemia. It participates in preadipocyte differentiation into adipocytes and in the hyperplasia and hypertrophy of mature adipocytes (61).

Visfatin is another adipokine related to abdominal obesity that increases pro-inflammatory factors in monocytes, promoting T lymphocyte activation. Correlation between visfatin concentration and insulin resistance, measured by the HOMA (Homeostatic Model Assessment for Insulin Resistance)-index was found in patients with ankylosing spondylitis, chronic inflammatory arthritis closely related to psoriatic arthritis (62). At the vascular level, visfatin upregulates vascular endothelial growth factor (VEGF) secretion and downregulates metalloproteins expression (63).

The increased cardiovascular risk in obese patients may be related to altered fibrinolysis. Adipocytes produce PAI-1, and obese patients have a high PAI level correlated with insulin resistance (64).

Both IL-6 and TNF-α promote insulin resistance inhibiting lipoprotein lipase (LPL), decreasing triglyceride hydrolysis into free fatty acids, and increasing triglyceride storage in adipocytes, resulting in larger fat cells (57). Besides, TNF-α inhibits preadipocytes conversion to mature adipocytes (65) and promotes atherosclerotic plaque formation, favoring leukocyte recruitment and activation, endothelial cell expression of adhesion molecules, and triggering arterial wall inflammatory cascade (30).

Unlike TNF-α, whose change in local production has not been confirmed, the expression of IL-6 in visceral fat was higher than in subcutaneous fat (30).

### Anti-inflammatory Adipokines

Adiponectin is exclusively synthesized and secreted by adipose tissue. Its protective role in the development of MetS and atherosclerosis has been suggested as it improves insulin sensitivity and fatty acid oxidation (63). Patients with chronic diseases show low adiponectin levels resulting from a decreased synthesis in adipose tissue after the increase in IL-6 and TNFα cytokines. A low adiponectin level is associated with insulin resistance, vasodilation, endothelial damage, and diastolic failure and with a high CRP level in obese patients (30). In addition, adiponectin receptor mRNA (AdipoR1/R2) expresses to a lesser extent as well (66). Adiponectin concentration was inversely correlated with triglycerides/HDL and total cholesterol/HDL cholesterol ratios, and with high fasting plasma glucose level in rheumatoid arthritis, the prototypical inflammatory arthritis. Therefore, in rheumatoid arthritis, low adiponectin level clustered with metabolic syndrome features implicated in accelerated atherosclerosis development (67).

Omentin, produced by the vascular stroma cells of the visceral adiposity, increases insulin sensitivity, stimulating insulin-mediated glucose uptake in human adipocytes (68).

### Adipose Tissue Changes in Obesity

In obese patients, adipokines synthesis and secretion are altered. Adipocytes are dysfunctional and increase the secretion of pro-inflammatory adipokines (69), triggering systemic mild chronic inflammation in the adipose tissue at first, then spreading away all through (57). Leptin secretion increases secondary to the increase in adipocyte lipid content. Though aimed to reduce caloric intake by acting at the central nervous system, leptin secretion leads to an imbalance at the adipose tissue. Accordingly, infiltration and accumulation of proinflammatory M1-type macrophages increase, replacing the normally found type 2 macrophages. Not only IL6 and TNF-α increase but insulin resistance develops as well. Accompanying M1 macrophages, proinflammatory cells (Th1, B2 B cells and ILC1) increase and anti-inflammatory immune cells (Tregs, Bregs, and ILC2) decrease in adipose tissue, perpetuating chronic inflammation (57). In sum, the adipose tissue drives an imbalance between anti-inflammatory and pro-inflammatory adipokines favoring the latter, increasing insulin resistance, propitiating diabetes, MetS, and cardiovascular disease.

Faced with this scene, two ways to improve the inflammatory state in adipose tissue and insulin sensitivity appear: weight loss and white adipose tissue gaining characteristics of brown adipose tissue in the so-called beiging process. The resulting beige or brite (brown in white) adipocytes (59) can burn lipids and restore normal insulin sensitivity, reducing the proinflammatory environment. Exposure to cold, β-adrenergic stimulation and some cytokines like IL33, IL13 and IL4 activate this mechanism.

In obesity, this process is slowed down, hampering insulin sensitivity restoration. Interleukin-17 is postulated to inhibit adipocyte differentiation and glucose absorption, and stem cell differentiation into adipocytes (57).

Although IL-17–producing T cells represent only 10% of the total immune cells in adipose tissue, the increase in fatty acids regulates the differentiation of CD4 + T cells into IL-17 T cells through acetyl Co-A carboxylase 1, increasing IL-17 T cells in obese patients, limiting the ability of fatty tissue to turn into brown tissue, decrease the inflammatory state and improve insulin sensitivity (57).

Obese patients have higher levels of IL-17 and IL-23 compared with normal weight counterparts unrelated to abdominal fat, insulin-resistance, or leptin level (70).

Presumably, IL-17 synthesized by visceral fat might favor vascular smooth muscle cells expression of eotaxin, related to carotid intima-media thickening as a sign of subclinical atherosclerosis (71).

### Bone Marrow-White Adipose Tissue, Adiponectin, and the Bone

Bone marrow is a substantial reservoir of white adipose tissue alongside visceral and subcutaneous white fat. Adipocytes stand for up to 70% of total human bone marrow (72). Marrow adipose tissue (MAT) contributes to local and systemic metabolic processes with adipokine secretion (73). Its relevance as a paracrine organ, and its increase when bone mass decreases, have encouraged hypotheses regarding its role in bone health and metabolism. Bone MAT releases adiponectin in response to caloric restriction, highlighting its relevance as an endocrine organ (74).

Contrary to expectations, PsA with high BMI values show high adiponectin levels. Far from improving metabolism as in other tissues, adiponectin seems to have pro-inflammatory properties at the joints, increasing damage. Adiponectin proved a biomarker of radiographic progression independent of metabolic status in rheumatoid arthritis patients (75–77).

Regarding the involved mechanisms, adiponectin might act on osteoblasts, reducing the new bone formation and on osteoclasts, increasing bone resorption (73). Adiponectin and leptin receptors are found in osteoblasts, osteoclasts, and osteoclast precursors. Ultimately, the real impact of these adipokines in bone remodeling is still controversial (73).

In addition, adiponectin induces the release of proinflammatory cytokines like IL-6, monocytic chemotactic protein 1 (MCP-1), prodegradative enzymes like metalloproteinases, and nitric oxide in chondrocytes. Synovial fibroblasts have adiponectin receptors whose stimulation also contributes to promoting a prodegradative microenvironment (78–80).

Different adiponectin isoforms might account for the anti-inflammatory and pro-inflammatory properties of adiponectin (81).

## Relationships and Change in Therapeutics

### Nonbiological Systemic Agents

Currently, peripheral joint compromise is initially treated with synthetic disease-modifying drugs like methotrexate, leflunomide, sulfasalazine, and cyclosporine. Acute episodes are often controlled with non-steroidal anti-inflammatory drugs (NSAIDs), which should be used with caution in patients with cardiovascular disease or cardiovascular risk factors. Similarly, small corticosteroids dose for as short as possible should be used (82).

Disease-modifying antirheumatic drugs (DMARDs) should have a positive impact on cardiovascular disease in PsA patients since their pharmacological action reduces systemic inflammation.

Methotrexate (MTX) is a folate antagonist that inhibits the activity of dihydrofolate reductase activity and is one of the most widely used synthetic drugs in PsA treatment. Unfortunately, its effects on the cardiovascular are diametrically opposed, and, eventually, its prolonged administration may increase serum homocysteine level (83) and cardiovascular risk in patients with previous hyperhomocysteinemia (84). Fortunately, increasing homocysteine levels by MTZ can be prevented with folic acid administration (83) while keeping the anti-inflammatory properties that reduce cardiovascular risk. Large observational studies have shown that MTX reduces cardiovascular disease incidence in PsA and rheumatoid arthritis patients. Low or moderate cumulative doses and concomitant use of folic acid have shown more beneficial than high cumulative doses (85). However, the risk of hospitalization for ischemic heart disease among people who received MTX was comparable to that of people receiving other non-biologic antipsoriatic drugs like oral retinoids, cyclosporine, azathioprine, and mycophenolate (86).

Regarding liver disease, it may beiatrogenic or due to the disease itself. Leflunomide and MTX can induce nonalcoholic steatohepatitis (NASH). NASH was more frequent in MTX-treated patients and in patients with PsA compared with rheumatoid arthritis (87). However, PsA patients treated with an anti–TNF-α/MTX association had a lower risk of liver fibrosis compared with those treated with MTX alone (87).

The presence of liver disease should be considered when selecting the PsA treatment (16). Hepatic steatosis, found in 28.1% of the studied sample, was an independent predictor for not achieving the MDA (hazard ratio [HR], 1.91; 95% confidence interval [CI], 1.04–3.38) as reported by a prospective study (88). Patients with liver disease should not be treated with MTX, leflunomide, sulfasalazine, and NSAIDs. Whenever these drugs are administered, careful monitoring of transaminases and liver function should be performed.

### Biological Agents

Proteins and/or their derivatives targeting specific molecular cascade steps involved in the pathophysiology of different diseases are defined as biological agents (89). According to their molecular structure, they are classified as recombinant human cytokines and growth factors, and fusion antibody proteins and monoclonal antibodies (89).

Fusion proteins are molecules formed by a naturally occurring receptor bound to an immunoglobulin structure. Monoclonal antibodies may be (a) chimeric antibodies, having 30% murine genes fused with human antibodies, (b) humanized antibodies, with 10% murine sequences, and (c) human antibodies derived solely from human immunoglobulin genes.

The development of biological drugs and small molecules pharmacologically active has expanded the therapeutic arsenal in PsA management. This has improved life quality and prognosis in patients refractory to methotrexate, sulfasalazine, leflunomide, or cyclosporin A, conventional immunosuppressive agents.

The complexity and systemic characteristics of PsA demand considering both the ongoing disease activity and extra-articular manifestations, and comorbidities before coming to a drug choice.

Evidence-based, obesity reflects mild chronic systemic inflammation. Worse, visceral fat might add to PsA intrinsic inflammation, secreting pro-inflammatory cytokines. As a result, it affects the therapeutic response and increases cardiovascular risk and the likelihood of expressing the disease in genetically predisposed subjects.

Abdominal obesity not only increases systemic inflammation in a positive feed-back cycle but is relevant to the therapeutic response.

#### Anti TNF α Agents

The TNF-α cytokine is key in PsA pathophysiology. Currently, five anti-TNFα molecules are available. Infliximab, adalimumab, and golimumab are monoclonal antibodies. Etanercept is a genetically manufactured dimeric protein resulting from the fusion of the soluble extracellular domain of human TNF-2α (TNFR2/p75) bound to the human IgG1 Fc domain. Certolizumab pegol is a humanized murine monoclonal antibody Fab fragment bound to two polyethylene glycol molecules.

Psoriatic arthritis patients treated with TNF-α underwent a larger decrease in IMTc, a marker of subclinical atherosclerotic disease, compared with those treated with synthetic disease-modifying drugs (6). However, obesity is a negative predictive factor for achieving minimum disease activity (MDA) in patients treated with biological therapies. Treatment with TNF-α inhibitors may induce weight gain in PsA patients (90).

In 2012, Di Minno et al. found that obesity was prevalent in patients not reaching the MDA end-point compared with those who did (64.0% versus 25.5%, p <0.001). Obesity was associated with an increased risk of not reaching MDA (HR = 4.90, 95% CI = 3.04–7.87, p<0.001). The HR for not reaching MDA was 3.98 (95% CI = 1.96–8.06, p< 0.001) and 5.40 (95% CI = 3.09–9.43, p<0.001) in subjects with BMI <30 kg/m^2^ and <30 to 35 kg/m^2^. In 98 subjects achieving MDA at 12 months of study, obesity was associated with a low probability of maintaining MDA at 24 months of follow-up (HR = 2.04, 95% CI = 1,015–3.61, p = 0.014) (91). In psoriatic arthritis, the disease activity index for PsA (DAPSA) score was associated with increased risk of subclinical atherosclerotic disease (92).

Assuming the negative effect of obesity in the disease’s control, the same group of researchers studied the effect of a low-calorie diet and weight loss in 126 obese patients starting anti–TNF-α administration. After adjusting for any other clinical and laboratory factors, the dietary intervention proved a strong and independent predictor of MDA achievement (93).

This has probably been related to drug elimination. Patients weighing over100 kg eliminate the drug 55% faster and have a 35% larger distribution volume, reaching lower minimum levels at the time of dosing (94).

In obese patients with metabolic syndrome, the TNF-α blockade may be less effective. The circulating T helper (Th) 17 level and IL-1 cells, elevated in obese patients, can predict the response inadequate to Anti TNF-α (95).

The Danish and Icelandic biological treatment registry included 1943 patients with PsA. Of these, 1271 (65%) had a BMI, with 408 (32%) obese. Obese patients had a higher disease activity and poor adherence to treatment, men in particular, with the median duration of Anti-TNF-α treatment of 2.5 years (95% CI = 1.7–3.2) in the obese vs 5.9 (4.1–7.7) in the non-obese. (P <0.01). Obesity came out as a risk factor for abandoning treatment [HR = 1.6 (95% CI = 1.3–2.0)] (96).

Six-month treatment with adalimumab increased insulin sensitivity and reduced CRP and retinol-binding protein-4 (RBP-4) in a prospective study of 29 patients with moderate-to-severe psoriasis (97, 98). These results were supported by a meta-analysis that included 38 randomized controlled trials (RCTs) with 18024 patients reporting adverse events in adults with plaque psoriasis who received at least one dose of biological therapy, conventional systematic therapy, or placebo. No risk differences for major cardiovascular events were associated with the use of AntiTNF-α (odds ratio [OR] = 0.67, 95% CI = 0. 10–4.63) (99).

#### Anti IL-17 Agents

Among the biological agents acting through IL-17 inhibition, we find secukinumab and ixekizumab, which are anti–IL-17A monoclonal antibodies. Secukinumab efficacy in obese patients is controversial. In a retrospective observational study including 136 patients with cutaneous psoriasis from 10 dermatology centers in Spain with a 52-week follow-up, secukinumab efficacy was lower in patients with a BMI above 30 (100).

There is scarce information on secukinumab efficacy in obese patients with psoriatic arthritis.

Recently, Pantano et al., carried out a prospective analysis of 100 patients with PsA, divided into those with a BMI above or below 25. After 6 months of treatment, overweight and obese patients had an even better response to secukinumab compared with normal weight patients. Analyzing serum IL-17 levels in 20 obese and 20 non-obese patients, higher serum levels of IL-17 were found in the former (101). This finding was in line with the literature since obesity promotes the expansion of IL17-producing T cells in adipose tissue, and higher levels of IL17 and IL23 were found in this study.

A recent retrospective study of 290 PsA patients, 310 PsC patients and 600 healthy controls found that obesity was more frequent in either PsC (36.5% vs. 22%, OR = 2.1, 95% CI = 1.5–2.8, p <0.01) or PsA (27.6% vs. 22%, OR = 1.4, 95% CI = 1.0–1.9, p <0.05) compared with controls with no inflammatory disease. Curiously, obesity was more frequent in PsC (36.5%) than in PsA (27.6%) (OR = 1.5, 95% CI = 1.1 to 2.1, p <0.05). A family history of PsA (OR = 3.6, 95% CI = 1.1–12.4), axial compromise (OR = 4.4, 95% CI = 1.0–15.4), and dyslipidemia (OR = 3.5, 95% CI = 1.5–8.6) were independently associated with obesity after correcting for probable confounding factors (102).

The effect of IL-17 on cardiovascular risk has been the subject of debate, likely due to its dual anti-atherogenic and pro-atherogenic action, depending on the inflammatory context (22).

An observational study including 195 secukinumab-treated patients with a 2-year follow-up reported that 2% of patients experienced a CV event (103). However, the safety data of the published meta-analysis did not risk differences in the risk of major cardiovascular events associated with the use of anti-IL-17A agents (secukinumab and ixekizumab) (OR = 1·00, 95% CI = 0·09–11·09) (99).

#### Anti-IL-12/IL-23

Ustekinumab is the first biological drug specifically targeted at IL-12/IL-23 approved to treat PsA. It binds to the p40 subunit, which is shared by IL-12 and IL-23, interfering with Th1 and Th17-dependent proinflammatory cytokines´ production (95).

Recent studies have shown that PsA patients´ response to ustekinumab was effective and sustained, regardless of body weight (104).

In a study of 79 patients diagnosed with cutaneous psoriasis, patients treated for 7 months with infliximab had an increase (P <0.001) in the mean BMI (2.1 ± 4.5%) and body weight. (2.5 ± 3.3 kg) compared with those treated with ustekinumab (0.1 ± 3.3%; 0.6 ± 1.1 kg) (105).

The pivotal, randomized, placebo-controlled, phase III PSUMMIT I trial not only confirmed the efficacy of ustexinumab in PsA but, unlike other biological agents, those with a bodyweight below or equal to 100 kg had higher response rates than those weighing over 100 kg. In a *post hoc* analysis by weight group, patients over 100 kg treated with the 90 mg dose showed a trend to higher American College of Rheumatology (ACR) and Psoriasis Area Severity Index (PASI) response rates than those treated with the 45-mg dose (106). Whether these differences are due to suboptimal dosing is to be determined (95).

An observational study of 50 patients with PsA, of whom 28% were obese, studied the efficacy of using ≥3 doses of ustekinumab. Fifty-four percent of patients treated with ustekinumab achieved an MDA response (107).

Regarding ustekinumab on cardiovascular risk, the debate is still open. Contrary to expectations, as IL-12 and IL-23 are found in atheroma plaques, the first data obtained from clinical studies using inhibitors reported a higher rate of major cardiovascular events in the ustekinumab group (108). However, later on, there was no increase in the number of events in the study, so the risk was even lower than in the general population (108).

Two meta-analyses have been carried out to assess the risk of major cardiovascular events (109, 110). Although one possible explanation has been that the methods used for data analysis were different, the results of both meta-analyses turned out contradictory (22). One of them reported no statistically significant differences in the risk of major cardiovascular events compared with placebo (OR = 4.48, 95% CI = 0.24–84.77, p = 0.32) (99)

Randomized long-term follow-up controlled trials are necessary to make recommendations (22).

## Conclusion

Psoriasis, a systemic disease entangling MetS, cardiovascular disease, and joint involvement, puts forth obesity and PsA as indirect indicators of disease severity (111). The high prevalence of MetS and its components in PsA patients stresses the importance of performing anamnesis, thorough physical examination, and carotid arteries’ Doppler evaluation, screening subclinical atherosclerotic disease. Counseling on lifestyle changes pointing to a well-balanced, healthy diet, smoking cessation, increasing physical activity, sleep care, and so on, is essential and should take part in their comprehensive assessment. Ultimately, non-classical factors’ influence on cardiovascular disease development should not be underestimated, demanding rigorous control of disease activity.

Provided the large available therapeutic arsenal, the choice of treatment should consider the individual characteristics, obesity, and metabolic syndrome, in particular.

Optimal care of PsA patients is a huge challenge for the treating physicians. Therapeutic management should not be limited to the care of the skin and joints. The systemic and inflammatory nature of the disease makes the search for comorbidities imperative. Adequate cardiovascular risk assessment and strict bodyweight control should be considered therapeutic objectives. These will not only modify the patient’s prognosis but influence therapeutic efficacy and have to be considered. In this way, the comprehensive management of the patient will be decisive in the choice of the therapeutic scheme.

## Author Contributions

SP, RK: writing—original draft. VC, EK: supervision. MO-L: review and editing—grammar, style, and language. FC: funding acquisition, writing—review. All authors contributed to the article and approved the submitted version.

## Funding

This work was supported by grant PICD 0031 (2016–2020) from FONCyT. Argentina.

## Conflict of Interest

The authors declare that the research was conducted in the absence of any commercial or financial relationships that could be construed as a potential conflict of interest.

## References

[1] Takeshita J Grewal S Langan SM Mehta NN Ogdie A Van Voorhees AS Psoriasis and comorbid diseases: Epidemiology. J Am Acad Dermatol (2017) 76(3):377–90. 10.1016/j.jaad.2016.07.064 PMC5731650 28212759

[2] Gladman DD Natural history of psoriatic arthritis. Baillieres Clin Rheumatol (1994) 8(2):379–94. 10.1016/S0950-3579(94)80024-3 8076393

[3] Caso F Chimenti MS Metabolic Syndrome and psoriatic arthritis: considerations for the clinician. Expert Rev Clin Immunol (2020) 16: (4):409–20. 10.1080/1744666X.2020.1740593 32149545

[4] Gladman DD Antoni C Mease P Clegg DO Nash P Psoriatic arthritis: epidemiology, clinical features, course, and outcome. Ann Rheum Dis (2005) 64 Suppl 2(Suppl 2):ii14–7. 10.1136/ard.2004.032482 PMC1766874 15708927

[5] Raimondo A Lembo S Di Caprio R Donnarumma G Monfrecola G Balato N Psoriatic cutaneous inflammation promotes human monocyte differentiation into active osteoclasts, facilitating bone damage. Eur J Immunol (2017) 47(6):1062–74. 10.1002/eji.201646774 28386999

[6] Ritchlin CT Colbert RA Gladman DD Psoriatic Arthritis. N Engl J Med (2017) 376(10):957–70. 10.1056/NEJMra1505557 28273019

[7] Giacomelli R Afeltra A Alunno A Bartoloni-Bocci E Berardicurti O Bombardieri M Guidelines for biomarkers in autoimmune rheumatic diseases - evidence based analysis. Autoimmun Rev (2019) 18(1):93–106. 10.1016/j.autrev.2018.08.003 30408582

[8] Kehl AS Corr M Weisman MH Review: Enthesitis: New Insights Into Pathogenesis, Diagnostic Modalities, and Treatment. Arthritis Rheumatol (2016) 68(2):312–22. 10.1002/art.39458 PMC5195265 26473401

[9] Gladman DD Ziouzina O Thavaneswaran A Chandran V Dactylitis in psoriatic arthritis: prevalence and response to therapy in the biologic era. J Rheumatol (2013) 40(8):1357–9. 10.3899/jrheum.130163 23818708

[10] Taylor W Gladman D Helliwell P Marchesoni A Mease P Mielants H CASPAR Study Group Classification criteria for psoriatic arthritis: development of new criteria from a large international study. Arthritis Rheum (2006) 54(8):2665–73. 10.1002/art.21972 16871531

[11] Tillett W Costa L Jadon D Wallis D Cavill C McHugh J The ClASsification for Psoriatic ARthritis (CASPAR) criteria–a retrospective feasibility, sensitivity, and specificity study. J Rheumatol (2012) 39(1):154–6. 10.3899/jrheum.110845 22089469

[12] Cañete JD Mease P The link between obesity and psoriatic arthritis. Ann Rheum Dis (2012) 71(8):1265–6. 10.1136/annrheumdis-2012-201632 22798633

[13] Kolliker Frers RA Cosentino V Tau J Kerzberg EM Urdapilleta A Chiocconi M Immune-Mediated Inflammation Promotes Subclinical Atherosclerosis in Recent-Onset Psoriatic Arthritis Patients without Conventional Cardiovascular Risk Factors. Front Immunol (2018) 9:139. 10.3389/fimmu.2018.00139 29535705 PMC5834432

[14] Icen M Crowson CS McEvoy MT Dann FJ Gabriel SE Maradit Kremers H Trends in incidence of adult-onset psoriasis over three decades: a population-based study. J Am Acad Dermatol (2009) 60(3):394–401. 10.1016/j.jaad.2008.10.062 19231638 PMC3028518

[15] Jensen P Skov L Psoriasis and Obesity. Dermatology (2016) 232(6):633–9. 10.1159/000455840 28226326

[16] Husni ME Comorbidities in Psoriatic Arthritis. Rheum Dis Clin North Am (2015) 41(4):677–98. 10.1016/j.rdc.2015.07.008 26476226

[17] Talotta R Atzeni F Sarzi-Puttini P Masala IF Psoriatic arthritis: From pathogenesis to pharmacologic management. Pharmacol Res (2019) 148:104394. 10.1016/j.phrs.2019.104394 31505253

[18] Rehaume LM Mondot S Aguirre de Cárcer D Velasco J Benham H Hasnain SZ ZAP-70 Genotype Disrupts the Relationship Between Microbiota and Host, Leading to Spondyloarthritis and Ileitis in SKG Mice. Arthritis Rheumatol (2014) 66(10):2780–92. 10.1002/art.38773 25048686

[19] McGonagle D Aydin SZ Tan AL The synovio-entheseal complex and its role in tendon and capsular associated inflammation. J Rheumatol Suppl (2012) 89:11–4. 10.3899/jrheum.120233 22751582

[20] McGonagle D Lories RJ Tan AL Benjamin M The concept of a “synovio-entheseal complex” and its implications for understanding joint inflammation and damage in psoriatic arthritis and beyond. Arthritis Rheum (2007) 56(8):2482–91. 10.1002/art.22758 17665450

[21] Dolcino M Lunardi C Ottria A Tinazzi E Patuzzo G Puccetti A Crossreactive autoantibodies directed against cutaneous and joint antigens are present in psoriatic arthritis. PLoS One (2014) 9(12):e115424. 10.1371/journal.pone.0115424 25514237 PMC4267814

[22] Caiazzo G Fabbrocini G Di Caprio R Raimondo A Scala E Balato N Psoriasis, Cardiovascular Events, and Biologics: Lights and Shadows. Front Immunol (2018) 9:1668. 10.3389/fimmu.2018.01668 30150978 PMC6099159

[23] Naldi L Chatenoud L Linder D Belloni Fortina A Peserico A Virgili AR Cigarette smoking, body mass index, and stressful life events as risk factors for psoriasis: results from an Italian case-control study. J Invest Dermatol (2005) 125(1):61–7. 10.1111/j.0022-202X.2005.23681.x 15982303

[24] Sommer DM Jenisch S Suchan M Christophers E Weichenthal M Increased prevalence of the metabolic syndrome in patients with moderate to severe psoriasis. Arch Dermatol Res (2006) 298(7):321–8. 10.1007/s00403-006-0703-z 17021763

[25] Flammer AJ Ruschitzka F Psoriasis and atherosclerosis: two plaques, one syndrome? Eur Heart J (2012) 33(16):1989–91. 10.1093/eurheartj/ehr425 22108835

[26] Scarpa R Caso F Costa L Peluso R Spanò A Lubrano E Psoriatic Disease: Clinical Staging. J Rheumatol Suppl (2015) 93:24–6. 10.3899/jrheum.150629 26523050

[27] Otero-Losada M Llambí HG Ottaviano G Cao G Müller A Azzato F Cardiorenal Involvement in Metabolic Syndrome Induced by Cola Drinking in Rats: Proinflammatory Cytokines and Impaired Antioxidative Protection. Mediators Inflamm (2016) 2016:5613056. 10.1155/2016/5613056 27340342 PMC4906210

[28] Boehncke WH Boehncke S Tobin A-M Kirby B The ‘psoriatic march’: a concept of how severe psoriasis may drive cardiovascular comorbidity. Exp Dermatol (2011) 20(4):303–7. 10.1111/j.1600-0625.2011.01261.x 21410760

[29] Kölliker Frers RA Bisoendial RJ Montoya SF Kerzkerg E Castilla R Takc PP Psoriasis and cardiovascular risk: Immune-mediated crosstalk between metabolic, vascular and autoimmune inflammation. IJC Metab Endocr (2015) 6:43–54. 10.1016/j.ijcme.2015.01.005

[30] Russolillo A Iervolino S Peluso R Lupoli R Di Minno A Pappone N Obesity and psoriatic arthritis: from pathogenesis to clinical outcome and management. Rheumatol (Oxford) (2013) 52(1):62–7. 10.1093/rheumatology/kes242 22989426

[31] Zhu TY Li EK Tam LS Cardiovascular risk in patients with psoriatic arthritis. Int J Rheumatol (2012) 2012:714321. 10.1155/2012/714321 22645614 PMC3356896

[32] Kimhi O Caspi D Bornstein NM Maharshak N Gur A Arbel Y Prevalence and risk factors of atherosclerosis in patients with psoriatic arthritis. Semin Arthritis Rheum (2007) 36(4):203–9. 10.1016/j.semarthrit.2006.09.001 17067658

[33] Gonzalez-Juanatey C Llorca J Amigo-Diaz E Dierssen T Martin J Gonzalez-Gay MA High prevalence of subclinical atherosclerosis in psoriatic arthritis patients without clinically evident cardiovascular disease or classic atherosclerosis risk factors. Arthritis Rheum (2007) 57(6):1074–80. 10.1002/art.22884 17665475

[34] Di Minno MN Ambrosino P Lupoli R Di Minno A Tasso M Peluso R Cardiovascular risk markers in patients with psoriatic arthritis: A meta-analysis of literature studies. Ann Med (2015) 47(4):346–53. 10.3109/07853890.2015.1031822 25953378

[35] Green A Shaddick G Charlton R Snowball J Nightingale A Smith C Modifiable risk factors and the development of psoriatic arthritis in people with psoriasis. Br J Dermatol (2020) 182(3):714–20. 10.1111/bjd.18227 31209855

[36] Eder L Abji F Rosen CF Chandran V Gladman DD The Association Between Obesity and Clinical Features of Psoriatic Arthritis: A Case-control Study. J Rheumatol (2017) 44(4):437–43. 10.3899/jrheum.160532 28202737

[37] Eder L Jayakar J Pollock R Pellett F Thavaneswaran A Chandran V Serum adipokines in patients with psoriatic arthritis and psoriasis alone and their correlation with disease activity. Ann Rheum Dis (2013) 72(12):1956–61. 10.1136/annrheumdis-2012-202325 23243196

[38] Feld J Nissan S Eder L Rahat MA Elias M Rimar D Increased Prevalence of Metabolic Syndrome and Adipocytokine Levels in a Psoriatic Arthritis Cohort. J Clin Rheumatol (2018) 24(6):302–7. 10.1097/RHU.0000000000000721 29708516

[39] Xue Y Jiang L Cheng Q Chen H Yu Y Lin Y Adipokines in psoriatic arthritis patients: the correlations with osteoclast precursors and bone erosions. PLoS One (2012) 7(10):e46740. 10.1371/journal.pone.0046740 23144698 PMC3483160

[40] Caso F Postiglione L Covelli B Ricciardone M Di Spigna G Formisano P Pro-inflammatory adipokine profile in psoriatic arthritis: results from a cross-sectional study comparing PsA subset with evident cutaneous involvement and subset “sine psoriasis”. Clin Rheumatol (2019) 38(9):2547–52. 10.1007/s10067-019-04619-w 31147798

[41] Dikbas O Tosun M Bes C Tonuk SB Aksehirli OY Soy M Serum levels of visfatin, resistin and adiponectin in patients with psoriatic arthritis and associations with disease severity. Int J Rheum Dis (2016) 19(7):672–7. 10.1111/1756-185X.12444 25196858

[42] Peters MJL Watt P Cherry L Welsh P Henninger E Dijkmans BAC Lack of effect of TNFalpha blockade therapy on circulating adiponectin levels in patients with autoimmune disease: results from two independent prospective studies. Ann Rheum Dis (2010) 69(9):1687–90. 10.1136/ard.2009.114207 19640853

[43] Fassio A Gatti D Gisondi P Girolomoni G Viapiana O Giollo A Effects of secukinumab on serum adipocytokines: preliminary data. Reumatismo (2017) 69(3):105–10. 10.4081/reumatismo.2017.953 28933132

[44] Chandran V Abji F Perruccio AV Gandhi R Li S Cook RJ Serum-based soluble markers differentiate psoriatic arthritis from osteoarthritis. Ann Rheum Dis (2019) 78(6):796–801. 10.1136/annrheumdis-2018-214737 30910989

[45] Colak S Omma A Sandikci SC Yucel C Omma T Turhan T Vaspin, neutrophil gelatinase-associated lipocalin and apolipoprotein levels in patients with psoriatic arthritis. Bratisl Lek Listy (2019) 120(1):65–9. 10.4149/BLL_2019_010 30685995

[46] Wagner CL Visvanathan S Elashoff M McInnes IB Mease PJ Krueger GG Markers of inflammation and bone remodelling associated with improvement in clinical response measures in psoriatic arthritis patients treated with golimumab. Ann Rheum Dis (2013) 72(1):83–8. 10.1136/annrheumdis-2012-201697 PMC3551220 22975755

[47] Johnson CM Fitch K Merola JF Han J Qureshi AA Li WQ Plasma levels of tumour necrosis factor-α and adiponectin can differentiate patients with psoriatic arthritis from those with psoriasis. Br J Dermatol (2019) 181(2):379–80. 10.1111/bjd.17700 30695115

[48] Chiricozzi A Raimondo A Lembo S Fausti F Dini V Costanzo A Crosstalk between skin inflammation and adipose tissue-derived products: pathogenic evidence linking psoriasis to increased adiposity. Expert Rev Clin Immunol (2016) 12(12):1299–308. 10.1080/1744666X.2016.1201423 27322922

[49] Paller AS Mercy K Kwasny MJ Choon SE Cordoro KM Girolomoni G Association of pediatric psoriasis severity with excess and central adiposity: an international cross-sectional study. JAMA Dermatol (2013) 149(2):166–76. 10.1001/jamadermatol.2013.1078 PMC3624889 23560297

[50] Love TJ Zhu Y Zhang Y Wall-Burns L Ogdie A Gelfand JM Obesity and the risk of psoriatic arthritis: a population-based study. Ann Rheum Dis (2012) 71(8):1273–7. 10.1136/annrheumdis-2012-201299 PMC3645859 22586165

[51] Li W Han J Qureshi AA Obesity and risk of incident psoriatic arthritis in US women. Ann Rheum Dis (2012) 71(8):1267–72. 10.1136/annrheumdis-2011-201273 PMC4183754 22562978

[52] Organization WH Overweight/obesity: overweight by country. Global Health Observatory Data Reposit (2008) 2013:2013.

[53] Otero-Losada ME Mc Loughlin S Rodríguez-Granillo G Müller A Ottaviano G Moriondo M Metabolic disturbances and worsening of atherosclerotic lesions in ApoE-/- mice after cola beverages drinking. Cardiovasc Diabetol (2013) 12:57. 10.1186/1475-2840-12-57 23547749 PMC3626849

[54] Kumari R Kumar S Kant R An update on metabolic syndrome: Metabolic risk markers and adipokines in the development of metabolic syndrome. Diabetes Metab Syndr (2019) 13(4):2409–17. 10.1016/j.dsx.2019.06.005 31405652

[55] Herrera MI Udovin LD Toro-Urrego N Kusnier CF Luaces JP Otero-Losada M Neuroprotection Targeting Protein Misfolding on Chronic Cerebral Hypoperfusion in the Context of Metabolic Syndrome. Front Neurosci (2018) 12:339. 10.3389/fnins.2018.00339 29904335 PMC5990610

[56] Lafontan M Berlan M Do regional differences in adipocyte biology provide new pathophysiological insights? Trends Pharmacol Sci (2003) 24(6):276–83. 10.1016/S0165-6147(03)00132-9 12823953

[57] Stolarczyk E Adipose tissue inflammation in obesity: a metabolic or immune response? Curr Opin Pharmacol (2017) 37:35–40. 10.1016/j.coph.2017.08.006 28843953

[58] Elmquist JK Ahima RS Maratos-Flier E Flier JS Saper CB Leptin activates neurons in ventrobasal hypothalamus and brainstem. Endocrinology (1997) 138(2):839–42. 10.1210/endo.138.2.5033 9003024

[59] Sanchez-Gurmaches J Guertin DA Adipocyte lineages: tracing back the origins of fat. Biochim Biophys Acta (2014) 1842(3):340–51. 10.1016/j.bbadis.2013.05.027 PMC3805734 23747579

[60] Pina T Genre F Lopez-Mejias R Armesto S Ubilla B Mijares V Relationship of leptin with adiposity and inflammation and resistin with disease severity in psoriatic patients undergoing anti-TNF-alpha therapy. J Eur Acad Dermatol Venereol (2015) 29(10):1995–2001. 10.1111/jdv.13131 25823684

[61] Helfer G Wu QF Chemerin: a multifaceted adipokine involved in metabolic disorders. J Endocrinol (2018) 238(2):R79–r94. 10.1530/JOE-18-0174 29848608 PMC6026924

[62] Miranda-Filloy JA López-Mejias R Genre F Carnero-López B Ochoa R Diaz de Terán T Leptin and visfatin serum levels in non-diabetic ankylosing spondylitis patients undergoing TNF-α antagonist therapy. Clin Exp Rheumatol (2013) 31(4):538–45. 23711190

[63] Kong Y Zhang S Wu R Su X Peng D Zhao M New insights into different adipokines in linking the pathophysiology of obesity and psoriasis. Lipids Health Dis (2019) 18(1):171. 10.1186/s12944-019-1115-3 31521168 PMC6745073

[64] Mertens I Van Gaal LF Obesity, haemostasis and the fibrinolytic system. Obes Rev (2002) 3(2):85–101. 10.1046/j.1467-789X.2002.00056.x 12120424

[65] Xu H Sethi JK Hotamisligil GS Transmembrane tumor necrosis factor (TNF)-alpha inhibits adipocyte differentiation by selectively activating TNF receptor 1. J Biol Chem (1999) 274(37):26287–95. 10.1074/jbc.274.37.26287 10473584

[66] Engin A Adiponectin-Resistance in Obesity. Adv Exp Med Biol (2017) 960:415–41. 10.1007/978-3-319-48382-5_18 28585210

[67] Gonzalez-Gay MA Llorca J Garcia-Unzueta MT Gonzalez-Juanatey C De Matias JM Martin J High-grade inflammation, circulating adiponectin concentrations and cardiovascular risk factors in severe rheumatoid arthritis. Clin Exp Rheumatol (2008) 26(4):596–603. 18799090

[68] Toussirot E Aubin F Dumoulin G Relationships between Adipose Tissue and Psoriasis, with or without Arthritis. Front Immunol (2014) 5:368. 10.3389/fimmu.2014.00368 25161652 PMC4129363

[69] Tang QQ Lane MD Adipogenesis: from stem cell to adipocyte. Annu Rev Biochem (2012) 81:715–36. 10.1146/annurev-biochem-052110-115718 22463691

[70] Sumarac-Dumanovic M Stevanovic D Ljubic A Jorga J Simic M Stamenkovic-Pejkovic D Increased activity of interleukin-23/interleukin-17 proinflammatory axis in obese women. Int J Obes (Lond) (2009) 33(1):151–6. 10.1038/ijo.2008.216 18982006

[71] Tarantino G Costantini S Finelli C Capone F Guerriero E La Sala N Is serum Interleukin-17 associated with early atherosclerosis in obese patients? J Transl Med (2014) 12:214. 10.1186/s12967-014-0214-1 25092442 PMC4256548

[72] Fazeli PK Horowitz MC MacDougald OA Scheller EL Rodeheffer MS Rosen CJ Marrow fat and bone–new perspectives. J Clin Endocrinol Metab (2013) 98(3):935–45. 10.1210/jc.2012-3634 PMC3590487 23393168

[73] Sulston RJ Cawthorn WP Bone marrow adipose tissue as an endocrine organ: close to the bone? Horm Mol Biol Clin Invest (2016) 28(1):21–38. 10.1515/hmbci-2016-0012 27149203

[74] Cawthorn WP Scheller EL Learman BS Parlee SD Simon BR Mori H Bone marrow adipose tissue is an endocrine organ that contributes to increased circulating adiponectin during caloric restriction. Cell Metab (2014) 20(2):368–75. 10.1016/j.cmet.2014.06.003 PMC4126847 24998914

[75] Meyer M Sellam J Fellahi S Kotti S Bastard JP Meyer O Serum level of adiponectin is a surrogate independent biomarker of radiographic disease progression in early rheumatoid arthritis: results from the ESPOIR cohort. Arthritis Res Ther (2013) 15(6):R210. 10.1186/ar4404 24314299 PMC3978925

[76] Giles JT van der Heijde DM Bathon JM Association of circulating adiponectin levels with progression of radiographic joint destruction in rheumatoid arthritis. Ann Rheum Dis (2011) 70(9):1562–8. 10.1136/ard.2011.150813 PMC3543946 21571734

[77] Klein-Wieringa IR van der Linden MP Knevel R Kwekkeboom JC van Beelen E Huizinga TW Baseline serum adipokine levels predict radiographic progression in early rheumatoid arthritis. Arthritis Rheum (2011) 63(9):2567–74. 10.1002/art.30449 21567382

[78] Lago R Gomez R Otero M Lago F Gallego R Dieguez C A new player in cartilage homeostasis: adiponectin induces nitric oxide synthase type II and pro-inflammatory cytokines in chondrocytes. Osteoarthritis Cartilage (2008) 16(9):1101–9. 10.1016/j.joca.2007.12.008 18261936

[79] Kusunoki N Kitahara K Kojima F Tanaka N Kaneko K Endo H Adiponectin stimulates prostaglandin E(2) production in rheumatoid arthritis synovial fibroblasts. Arthritis Rheum (2010) 62(6):1641–9. 10.1002/art.27450 20222108

[80] Ehling A Schäffler A Herfarth H Tarner IH Anders S Distler O The potential of adiponectin in driving arthritis. J Immunol (2006) 176(7):4468–78. 10.4049/jimmunol.176.7.4468 16547285

[81] Tilg H Moschen AR Adipocytokines: mediators linking adipose tissue, inflammation and immunity. Nat Rev Immunol (2006) 6(10):772–83. 10.1038/nri1937 16998510

[82] Agca R Heslinga SC EULAR recommendations for cardiovascular disease risk management in patients with rheumatoid arthritis and other forms of inflammatory joint disorders: 2015/2016 update. Ann Rheum Dis (2017) 76: (1):17–28. 10.1136/annrheumdis-2016-209775 27697765

[83] Tiftikci A Ozdemir A Tarcin O Tarcin O Inanc N Sahinoglu S Influence of serum folic acid levels on plasma homocysteine concentrations in patients with rheumatoid arthritis. Rheumatol Int (2006) 26(3):191–4. 10.1007/s00296-004-0546-x 15645233

[84] Lin X Meng X Song Z Homocysteine and psoriasis. Biosci Rep (2019) 39(11):BSR20190867. 10.1042/BSR20190867 31670376 PMC6879356

[85] Prodanovich S Ma F Taylor JR Pezon C Fasihi T Kirsner RS Methotrexate reduces incidence of vascular diseases in veterans with psoriasis or rheumatoid arthritis. J Am Acad Dermatol (2005) 52(2):262–7. 10.1016/j.jaad.2005.02.002 15692471

[86] Chen YJ Chang Y-T Shen J-L Chen T-T Wang C-B Chen C-M Association between systemic antipsoriatic drugs and cardiovascular risk in patients with psoriasis with or without psoriatic arthritis: a nationwide cohort study. Arthritis Rheum (2012) 64(6):1879–87. 10.1002/art.34335 22161801

[87] Seitz M Reichenbach S Möller B Zwahlen M Villiger PM Dufour J-F Hepatoprotective effect of tumour necrosis factor α blockade in psoriatic arthritis: a cross-sectional study. Ann Rheum Dis (2010) 69(6):1148–50. 10.1136/ard.2009.116194 19854710

[88] Di Minno MND Iervolino S Peluso R Russolillo A Lupoli R Scarpa R Hepatic Steatosis and Disease Activity in Subjects with Psoriatic Arthritis Receiving Tumor Necrosis Factor-α Blockers. J Rheumatol (2012) 39(5):1042–6. 10.3899/jrheum.111391 22422493

[89] Balato A Scala E Balato N Caiazzo G Di Caprio R Monfrecola G Biologics that inhibit the Th17 pathway and related cytokines to treat inflammatory disorders. Expert Opin Biol Ther (2017) 17(11):1363–74. 10.1080/14712598.2017.1363884 28791896

[90] Renzo LD Saraceno R Schipani C Rizzo M Bianchi A Noce A Prospective assessment of body weight and body composition changes in patients with psoriasis receiving anti-TNF-α treatment. Dermatologic Ther (2011) 24(4):446–51. 10.1111/j.1529-8019.2011.01439.x 21910803

[91] di Minno MN Peluso R Iervolino S Lupoli R Russolillo A Scarpa R Obesity and the prediction of minimal disease activity: a prospective study in psoriatic arthritis. Arthritis Care Res (Hoboken) (2013) 65(1):141–7. 10.1002/acr.21711 22514189

[92] Palmou-Fontana N Martínez-Lopez D Corrales A Rueda-Gotor J Genre F Armesto S Disease Activity Influences Cardiovascular Risk Reclassification Based on Carotid Ultrasound in Patients with Psoriatic Arthritis. J Rheumatol (2020) 47(9):1344–53. 10.3899/jrheum.190729 31732555

[93] Di Minno MN Peluso R Iervolino S Russolillo A Lupoli R Scarpa R Weight loss and achievement of minimal disease activity in patients with psoriatic arthritis starting treatment with tumour necrosis factor α blockers. Ann Rheum Dis (2014) 73(6):1157–62. 10.1136/annrheumdis-2012-202812 PMC4033114 23771989

[94] Tiberio R Graziola F Miglino B Veronese F Annali G Savoia P Secukinumab for Psoriasis in Obese Patients: Minireview and Clinical Experience. Case Rep Dermatol (2019) 11(Suppl 1):29–36. 10.1159/000501990 31662736 PMC6816128

[95] Costa L Ramonda R Ortolan A Favero M Foti R Visalli E Psoriatic arthritis and obesity: the role of anti-IL-12/IL-23 treatment. Clin Rheumatol (2019) 38(9):2355–62. 10.1007/s10067-019-04663-6 31264033

[96] Højgaard P Glintborg B Kristensen LE Gudbjornsson B Love TJ Dreyer L The influence of obesity on response to tumour necrosis factor-α inhibitors in psoriatic arthritis: results from the DANBIO and ICEBIO registries. Rheumatol (Oxford) (2016) 55(12):2191–9. 10.1093/rheumatology/kew326 27651526

[97] Pina T Armesto S Lopez-Mejias R Genre F Ubilla B Gonzalez-Lopez MA Anti-TNF-α therapy improves insulin sensitivity in non-diabetic patients with psoriasis: a 6-month prospective study. J Eur Acad Dermatol Venereol (2015) 29(7):1325–30. 10.1111/jdv.12814 25353352

[98] Pina T Genre F Lopez-Mejias R Armesto S Ubilla B Mijares V Anti-TNF-α therapy reduces retinol-binding protein 4 serum levels in non-diabetic patients with psoriasis: a 6-month prospective study. J Eur Acad Dermatol Venereol (2016) 30(1):92–5. 10.1111/jdv.13005 25650695

[99] Rungapiromnan W Yiu ZZN Warren RB Griffiths CEM Ashcroft DM Impact of biologic therapies on risk of major adverse cardiovascular events in patients with psoriasis: systematic review and meta-analysis of randomized controlled trials. Br J Dermatol (2017) 176(4):890–901. 10.1111/bjd.14964 27518205 PMC5412670

[100] Notario J Deza G Vilarrasa E Valentí F Muñoz C Mollet J Treatment of patients with plaque psoriasis with secukinumab in a real-life setting: a 52-week, multicenter, retrospective study in Spain. J Dermatolog Treat (2019) 30(5):424–9. 10.1080/09546634.2018.1528000 30244618

[101] Pantano I Iacono D Favalli EG Scalise G Costa L Caso F Secukinumab efficacy in patients with PsA is not dependent on patients’ body mass index. Ann Rheum Dis (2020), pii:annrheumdis-2020-217251. 10.1136/annrheumdis-2020-217251 32169970

[102] Queiro R Lorenzo A Tejón P Coto P Pardo E Obesity in psoriatic arthritis: Comparative prevalence and associated factors. Medicine (Baltimore) (2019) 98(28):e16400. 10.1097/MD.0000000000016400 31305449 PMC6641803

[103] Egeberg A Ottosen MB Gniadecki R Broesby-Olsen S Dam TN Bryld LE Safety, efficacy and drug survival of biologics and biosimilars for moderate-to-severe plaque psoriasis. Br J Dermatol (2018) 178(2):509–19. 10.1111/bjd.16102 29094341

[104] Iannone F Santo L Bucci R Semeraro A Carlino G Paoletti F Drug survival and effectiveness of ustekinumab in patients with psoriatic arthritis. Real-life data from the biologic Apulian registry (BIOPURE). Clin Rheumatol (2018) 37(3):667–75. 10.1007/s10067-018-3989-2 29411182

[105] Gisondi P Conti A Galdo G Piaserico S De Simone C Girolomoni G Ustekinumab does not increase body mass index in patients with chronic plaque psoriasis: a prospective cohort study. Br J Dermatol (2013) 168(5):1124–7. 10.1111/bjd.12235 23320916

[106] Kavanaugh A Puig L Gottlieb AB Ritchlin C Li S Wang Y Maintenance of Clinical Efficacy and Radiographic Benefit Through Two Years of Ustekinumab Therapy in Patients With Active Psoriatic Arthritis: Results From a Randomized, Placebo-Controlled Phase III Trial. Arthritis Care Res (2015) 67(12):1739–49. 10.1002/acr.22645 PMC5063124 26097039

[107] Queiro R Brandy A Rosado MC Lorenzo A Coto P Carriles C Minimal Disease Activity and Patient-Acceptable Symptom State in Psoriatic Arthritis: A Real-World Evidence Study With Ustekinumab. JCR: J Clin Rheumatol (2018) 24(7):381–4. 10.1097/RHU.0000000000000751 29509560

[108] Reich K Langley RG Lebwohl M Szapary P Guzzo C Yeilding N Cardiovascular safety of ustekinumab in patients with moderate to severe psoriasis: results of integrated analyses of data from phase II and III clinical studies. Br J Dermatol (2011) 164(4):862–72. 10.1111/j.1365-2133.2011.10257.x 21332467

[109] Tzellos T Kyrgidis A Zouboulis CC Re-evaluation of the risk for major adverse cardiovascular events in patients treated with anti-IL-12/23 biological agents for chronic plaque psoriasis: a meta-analysis of randomized controlled trials. J Eur Acad Dermatol Venereol (2013) 27(5):622–7. 10.1111/j.1468-3083.2012.04500.x 22404103

[110] Ryan C Leonardi CL Krueger JG Kimball AB Strober BE Gordon KB Association between biologic therapies for chronic plaque psoriasis and cardiovascular events: a meta-analysis of randomized controlled trials. JAMA (2011) 306(8):864–71. 10.1001/jama.2011.1211 21862748

[111] Gladman DD Callen JP Early-onset obesity and risk for psoriatic arthritis. JAMA (2010) 304(7):787–8. 10.1001/jama.2010.1162 20726050

